# Cardiac structure and function in elite female athletes: A systematic review and meta‐analysis

**DOI:** 10.14814/phy2.15141

**Published:** 2021-12-11

**Authors:** Robyn Bryde, Andres I. Applewhite, Abd Moain Abu Dabrh, Bryan J. Taylor, Michael G. Heckman, Sara E. Filmalter, George Pujalte, Carlos Rojas, Alexander J. Heckman, Tara J. Brigham, Larry J. Prokop, Brian P. Shapiro

**Affiliations:** ^1^ Department of Cardiovascular Diseases Mayo Clinic Jacksonville Florida USA; ^2^ Department of Family Medicine Mayo Clinic Jacksonville Florida USA; ^3^ Division of General Internal Medicine Integrative Medicine and Health Mayo Clinic Jacksonville Florida USA; ^4^ Biomedical Statistics and Informatics Mayo Clinic Jacksonville Florida USA; ^5^ Division of Sports Medicine Department of Orthopedics Mayo Clinic Jacksonville Florida USA; ^6^ Department of Radiology Mayo Clinic Jacksonville Florida USA; ^7^ Library Public Services Mayo Clinic Jacksonville Florida USA; ^8^ Library Public Services Mayo Clinic Rochester Minnesota USA

**Keywords:** athlete's heart, cardiac magnetic resonance imaging, female athletes, right ventricular enlargement

## Abstract

We conducted a meta‐analysis to synthesize the best available evidence comparing cardiac biventricular structure and function using cardiac magnetic resonance imaging (CMR) and transthoracic echocardiography (TTE) in elite female athletes and healthy controls (HC). Chronic exposure to exercise may induce cardiac chamber enlargement as a means to augment stroke volume, a condition known as the “athlete's heart.” These changes have not been clearly characterized in female athletes. Multiple databases were searched from inception to June 18, 2019. Outcomes of interest included left ventricular (LV) and right ventricular (RV) dimensional, volumetric, mass, and functional assessments in female athletes. Most values were indexed to body surface area. The final search yielded 22 studies, including 1000 female athletes from endurance, strength, and mixed athletic disciplines. CMR‐derived LV end‐diastolic volume (LVEDV) and RV end‐diastolic volume (RVEDV) were greater in endurance athletes (EA) versus HC (17.0% and 18.5%, respectively; both *p* < 0.001). Similarly, TTE‐derived LVEDV and RVEDV were greater in EA versus HC (16.8% and 28.0%, respectively; both *p* < 0.001). Both LVEF and RVEF were lower in EA versus HC, with the most pronounced difference observed in RVEF via TTE (9%) (*p* < 0.001). LV stroke volume was greater in EA versus HC via both CMR (18.5%) and TTE (13.2%) (both *p* < 0.05). Few studies reported data for the mixed athlete (MA) population and even fewer studies reported data for strength athletes (SA), therefore a limited analysis was performed on MA and no analysis was performed on SA. This evidence‐synthesis review demonstrates the RV may be more susceptible to ventricular enlargement. General changes in LV and RV structure and function in female EA mirrored changes observed in male counterparts. Further studies are needed to determine if potential adverse outcomes occur secondary to these changes.

## Abstract 2

This article focuses on the morphological and physiological effects of endurance‐based exercise on the female heart. Much literature has been described in males, but this data features data that mirrors that of men and particularly focuses on left and right ventricular function using established and advanced imaging modalities.

## 1 INTRODUCTION

Chronic exposure to exercise leads to structural and functional cardiac adaptations, most notably with greater chamber dimensions and wall thickness (Maron & Pelliccia, 2006; Morganroth et al., 1975; Pelliccia et al., 1991, 1999). These changes are commonly referred to as the “athlete's heart” (Maron, 1986; Maron et al., 1995; Rost, 1982). While enlargement in response to exercise may be purely physiologic, such findings are also apparent in and may mimic pathological conditions such as dilated cardiomyopathy, left ventricular noncompaction, hypertrophic cardiomyopathy, and arrhythmogenic right ventricular cardiomyopathy (ARVC; Elliott et al., 2008). The current standards for normal cardiac structure and function have been outlined for both cardiac magnetic resonance imaging (CMR) and transthoracic echocardiography (TTE) but do not include reference values for athletes in general (Lang et al., 2006; Rudski et al., 2010). Evaluation of the effects of short‐term exercise interventions and male athlete's ventricular structure and function with CMR and TTE has been reported in previously published systematic reviews and meta‐analysis’; however, information specifically on female athletes remains sparse (D'Ascenzi et al., 2017, 2018; Diaz‐Canestro & Montero, 2020; Pluim et al., 2000; Utomi et al., 2013).

There has been a significant increase in the number of females participating in sports since the 1972 Title IX enactment of regulations allowing equal opportunity for athletic participation across sexes in the United States (O’Reilly et al., 2020). Since these regulations have become effective, women collegiate athletics have gone from less than 32,000 to more than 200,000 participants, and the number of females participating in high school athletics has increased from 300,000 to about three million (Kennedy, 2010; Koller, 2010). At the Olympic level, female athletes account for 50% of participants. The Tokyo 2021 Olympics are projecting 49% female participation which is markedly higher than that observed in the 1984 Olympics with only 23% female athletes (International Olympic Committee, 2019). Beyond these organized settings, overall physical activity, including female participation in recreational sports, has increased in many high‐income countries within the past few decades (Hallal et al., 2012).

With female participation in athletic activities at an all‐time high, it is crucial to understand the structural and functional cardiovascular adaptations to exercise in a similar manner as previously done for their male counterparts. Therefore, this systematic review and meta‐analysis were conducted to characterize biventricular structure and function in elite female athletes using CMR and TTE. This work also provides evidence of reported normal values for female endurance athletes (EA) and highlights quantitative differences across both imaging modalities.

## 2 METHODS

We followed a priori protocol that followed the reporting of this review, which follows the Preferred Reporting Items for Systematic Reviews and Meta‐Analyses (PRISMA) statement (McInnes et al., 2018).

### 2.1 Study eligibility

To better define cardiac structural changes in response to chronic exercise, we included comparative and non‐comparative studies that evaluated “elite” female athletes. Because no universal definition exists for what comprises an “elite athlete,” we focused on studies of athletes competing at the national or international level (collegiate or Olympic athletes) and/or highly trained recreational athletes (triathletes, marathon runners, etc.). Athletes were assigned to specific sport categories based on the static and dynamic components of the activities they participated in according to the Mitchell classification of sports (Mitchell et al., 2005). Endurance sports are characterized as high‐dynamic, low‐static, and mixed sports are characterized as high‐dynamic, high‐static, and strength sports are defined as high‐static and low‐dynamic. We included female athletes and matched controls between the ages of 18–55 that received CMR and/or TTE. Studies included in this review excluded subjects with pathological conditions, congenital defects, and history of cardiac enlargement and/or arrhythmia. We also excluded studies evaluating cardiac adaptations in scuba divers and studies that did not report data on the RV (right ventricle). There were no limits on the follow‐up period.

### 2.2 Search strategy

Medical librarians designed and conducted a comprehensive search with input from the study's principal investigator. This search strategy was not limited by date of publication, sample size, or language. The search included several databases from the time of their inception to June 18, 2019. The databases included Ovid MEDLINE Epub Ahead of Print, Ovid Medline In‐Process & Other Non‐Indexed Citations, Ovid MEDLINE, Ovid EMBASE, Ovid Cochrane Central Register of Controlled Trials, Ovid Cochrane Database of Systematic Reviews, and Scopus. Controlled vocabulary supplemented with keywords was used to search for studies of functional capacity in elite female athletes measured by CMR and TTE. The actual strategy is available from the reprint author.

### 2.3 Outcomes definition

We extracted primary outcomes data pertinent to left ventricle (LV) and RV structural and functional measurements ascertained by using functional parameters through CMR and TTE. Values other than those expressed by a percentage were indexed to body surface area (BSA; m^2^). Data included LV end‐diastolic volume (LVEDV ml/m^2^), LV end‐systolic volume (LVESV; ml/m^2^), LV end‐diastolic diameter (LVEDD; mm/m^2^), LV end‐systolic diameter (LVESD; mm/m^2^), LV mass (LVM; g/m^2^), LV wall thickness (mm/m^2^), RV end‐diastolic area (RVEDA; cm^2^/m^2^), RV end‐systolic area (RVESA; cm^2^/m^2^), RV end‐diastolic volume (RVEDV; ml/m^2^), RV end‐systolic volume (RVESV; ml/m^2^), and RV mass (RVM; g/m^2^). Volumetric assessment of the RV by TTE was obtained with three‐dimensional (3D) technology in selected studies. Secondary outcomes included assessment of ejection fraction (EF; %), stroke volume (SV; ml/m^2^), tricuspid annular plane systolic excursion (TAPSE; mm), and fractional area change (FAC; %).

### 2.4 Data screening and extraction

Reviewers screened each study using standardized forms. Conflicts were reconciled first among initial reviewers (RB, AA) or senior investigators (BS, AMAD). When a study was deemed eligible, the reviewers moved it to data extraction, determined its methodological quality, and collected descriptive and outcome data. Reviewers extracted data on patient demographics and relevant baseline characteristics (e.g., sample size, age, race), study design and variable, and outcomes of interest. All steps of data screening and abstraction were conducted blindly, independently, and in duplicate (by two reviewers).

### 2.5 Risk of bias assessment and quality of evidence

We used the Newcastle‐Ottawa Scale (NOS) for cohort and case‐control studies. Due to the non‐comparative nature of the available studies, we used a modified NOS in which we discounted the comparability criteria, thus assessment of risk of bias (methodological quality assessment) focused on outcome ascertainment, cohort selection, and attrition (Mohammed et al., 2017). Control arms of comparative studies were considered as case series for this purpose. We used the Joanna Briggs Institute (JBI) Critical Appraisal Checklist to assess the quality of cross‐sectional (prevalence) or case series studies (Munn et al., 2014). We graded the strength of evidence using the Grading of Recommendations Assessment, Development, and Evaluation (GRADE) approach (Mohammed et al., 2017).

### 2.6 Statistical analysis

Comparisons of outcomes between EA and healthy controls (HC), between mixed athletes (MA) and HC, and between EA and MA were made using a random effects meta‐analysis for continuous outcome variables (DerSimonian & Laird, 1986). Separately for each pair‐wise comparison made between groups, mean differences, and 95% confidence intervals (CIs) were estimated for each outcome. Between‐study heterogeneity in mean differences was examined by estimating the *I* ^2^ statistic, which measures the proportion of variation in mean differences between studies due to heterogeneity beyond chance (Higgins & Thompson, 2002). After applying a Bonferroni correction for multiple testing, *p* < 0.0016 (comparisons between EA and controls), <0.0029 (comparisons between MA and controls), and <0.0063 (comparisons between EA and MA) were considered statistically significant. All statistical tests were two‐sided. Statistical analyses were performed using R Statistical Software (version 3.4.2; R Foundation for Statistical Computing).

## 3 RESULTS

There were a total of 1734 results from the literature search with 22 studies meeting inclusion criteria (D’Ascenzi et al., 2017; Doronina et al., 2018; Hedman et al., 2015; Henriksen et al., 1999; Kooreman et al., 2019; Kramer et al., 2013; Lakatos et al., 2018; Leischik & Spelsberg, 2014; Leischik et al., 2016; Luijkx, Cramer, et al., 2012; Luijkx, Velthuis, et al., 2012; Malmgren et al., 2015; Mangold et al., 2013; Petersen et al., 2006; Prakken et al., 2010, 2011; Sanz‐de la Garza et al., 2017; Steding‐Ehrenborg et al., 2016; Stolt et al., 2000; Venckunas et al., 2016; Zeldis et al., 1978). Of the studies that met the inclusion criteria, three were case series (Kooreman et al., 2019; Leischik & Spelsberg, 2014; Mangold et al., 2013), 16 were cross sectional (Doronina et al., 2018; Hedman et al., 2015; Henriksen et al., 1999; Kramer et al., 2013; Lakatos et al., 2018; Leischik et al., 2016; Luijkx, Cramer, et al., 2012; Luijkx, Velthuis, et al., 2012; Malmgren et al., 2015; Petersen et al., 2006; Prakken et al., 2010, 2011; Sansonio de Morais et al., 2017; Sanz‐de la Garza et al., 2017; Stolt et al., 2000; Zeldis et al., 1978), and three were cohort (with historical control) studies (D’Ascenzi et al., 2017; Steding‐Ehrenborg et al., 2016; Venckunas et al., 2016). A total of 529 HC, 501 EA, 421 MA, and 78 strength athletes (SA) results were included. Table 1 details results of the literature search and lists demographic information and sports classification for each study. Results of the risk of bias assessment are included in Appendix S1. The quality of evidence was downgraded to low, mainly due to indirectness (heterogeneity) and overall higher risk of bias due to design limitations of the included studies.

**TABLE 1:** Demographic characteristics of CMR and TTE studies assessing cardiac structure and function in female athletes

First author (year)	Sport category	*n*	Age (*SD*)	HR (*SD*)	BSA (*SD*)	Training regimen
Kooreman et al. (2019)	Control	31	19 (1)	—	1.7 (0.2)	<2 h/week
	Endurance	35	19 (1)	—	1.7 (0.1)	—
	Strength	37	19 (1)	—	1.8 (0.2)	—
Lakatos et al. (2018)	Control	20	19.5 (2.3)	84.2 (19.2)	1.6 (0.1)	<3 h/week
	Endurance	30	19 (3.7)	71.7 (12.1)	1.8 (0.1)	17 ± 6 h/week
Doronina et al. (2018)	Control	15	23 (2)	82 (7)	1.6 (0.1)	<3 h/week
	Endurance	15	24 (4)	69 (14)	1.8 (0.1)	24 ± 8 h/week
	Strength	15	24 (3)	63 (9)	1.6 (0.1)	12 ± 2 h/week
Henriksen et al. (1999)	Control	42	24.4 (3.3)	64 (8.6)	1.7 (0.14)	—
	Endurance	32	19.7 (2.4)	56 (9.5)	1.65 (0.09)	300–600 h/year
Sansonio de Morais et al. (2017)	Control	22	22.7 (3.4)	—	1.6 (0.2)	<2 h/week
	Endurance	22	23.3 (4.5)	—	1.7 (0.2)	20 h/week
D’Ascenzi et al. (2017)	Mixed	363	24 (6)	—	1.7 (0.2)	—
Leischik et al. (2016)	Control	37	31.2 (6.3)	69.7 (12.3)	1.81 (0.16)	—
	Endurance	33	34.3 (8.1)	61.6 (8.6)	1.7 (0.13)	More than 9 h/week
Venckunas et al. (2016)	Control	20	23 (2.3)	—	1.68 (0.1)	<2 h/week
	Strength	11	24.7 (2.8)	—	1.65 (0.09)	5–12 h/week
Malmgren et al. (2015)	Control	33	23 (2)	70 (13)	1.7 (0.1)	<2 h/week
	Endurance	33	20 (2)	56 (8)	1.9 (0.1)	10.8 ± 2.3 h/week
Hedman et al. (2015)	Control	48	21 (2)	71 (10)	1.63 (0.09)	—
	Endurance	46	21 (2)	54 (8)	1.69 (0.1)	13 ± 5 h/week
Leischik and Spelsberg (2014)	Endurance	33	34 (8.1)	—	1.7 (0.13)	15.5 ± 3.3 h/week
Mangold et al. (2013)	Endurance	23	28.4 (8.5)	58 (7.3)	1.7 (0.2)	12.8 ± 3 h/week
Luijkx, Cramer, et al. (2012)	Control	58	26 (5.8)	61 (10)	1.74 (0.11)	<3 h/week
	Endurance	51	23 (4.9)	56 (7.8)	1.74 (0.11)	17 ± 6.6 h/week
	Mixed	24	27 (4.4)	54 (7.7)	1.83 (0.13)	17 ± 6.6 h/week
	Strength	15	26 (4.8)	53 (6.9)	1.88 (0.21)	17 ± 6.6 h/week
Prakken et al. (2011)	Control	25	28 (6.2)	68 (9.7)	1.7 (0.1)	2.1 ± 1.1 h/week
	Endurance	24	26 (4.3)	55 (8.4)	1.9 (0.2)	22 ± 4.6 h/week
Prakken et al. (2010)	Control	58	26 (5.9)	67 (9.8)	1.7 (0.1)	<3 h/week
	Endurance	33	25 (4.2)	57 (8.6)	1.9 (0.2)	More than 18 h/week
Petersen et al. (2006)	Control	17	26 (3)	66 (10)	1.72 (0.16)	—
	Endurance	20	24 (4)	56 (9)	1.79 (0.1)	19 ± 0.5 h/week
Stolt et al. (2000)	Control	15	26 (5)	68 (13)	1.6 (0.1)	<2 h/week
	Endurance	30	24 (4)	50 (10)	1.7 (0.1)	10 ± 2 h/week
Zeldis et al. (1978)	Control	25	22.1 (0.1)	71.4 (2.8)	1.57 (0.02)	—
	Mixed	10	20.1 (0.5)	58.6 (2.8)	1.62 (0.02)	—
Zeldis et al. (1978)	Control	13	25.5 (4.2)	73.6 (11.6)	1.6 (0.1)	<3 h/week
	Endurance	13	25.1 (4.7)	59.9 (4)	1.84 (0.14)	12 ± 2 h/week
Sanz‐de la Garza et al. (2017)	Control	20	36.9 (4.6)	74.9 (7.6)	1.62 (0.11)	—
	Endurance	20	37.4 (6.3)	59.9 (5.1)	1.6 (0.1)	3.2 ± 1.4 h/week
Steding‐Ehrenborg et al. (2016)	Control	6	33 (10)	60 (9)	1.75	—
	Endurance	8	25 (5)	51 (3)	1.62	—
Luijkx, Velthuis, et al. (2012)	Control	24	38.2 (13)	—	1.75 (0.11)	<3 h/week
	Mixed	24	38.1 (14)	—	1.79 (0.12)	More than 9 h/week

**FIGURE 1:**
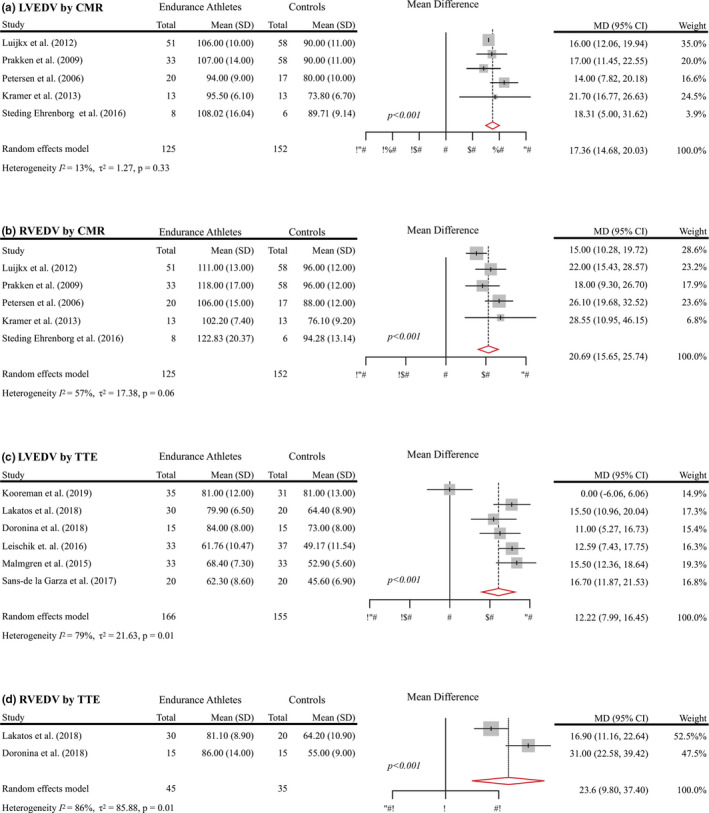
Forest plot showing mean differences between (EA vs. HC) in LVEDV indexed to BSA (ml/m^2^) using CMR (a), RVEDV indexed to BSA (ml/m^2^) using CMR (b), LVEDV indexed to BSA (ml/m^2^) using TTE (c), and RVEDV indexed to BSA (ml/m^2^) using TTE (d). The size of the square corresponds to the weight of each study. The diamonds and their width represent the pooled mean difference (MD) and the 95% confidence intervals (95% CIs), respectively. BSA, body surface area; CMR, cardiac magnetic resonance imaging; LVEDV, left ventricular end‐diastolic volume; LVESV, left ventricular end‐systolic volume; RVEDV, right ventricular end‐diastolic volume; RVESV, right ventricular end‐systolic volume; TTE, transthoracic echocardiography

A meta‐analysis was performed on EA and HC using CMR and TTE and is shown in Table 2. A limited analysis was performed on MA and HC and on EA and MA and is included in Appendices S2 and S3, respectively. A forest plot for LVEDV and RVEDV mean differences between groups and imaging modality are shown in Figure 1. Forest plots for additional variables are included in Appendix [Link], [Link].

**TABLE 2:** Meta‐analysis of endurance athletes and healthy controls

Outcome	Number of studies	Endurance sample size	Controls sample size	Endurance: mean value across studies	Controls: mean value across studies	Mean difference—endurance minus controls (95% CI)	*p*‐value	*I* ^2^
CMR								
LVEDV (ml/m^2^)	5	125	152	102.10	84.70	17.36 (14.68, 20.03)	<0.001	0.13
LVESV (ml/m^2^)	4	117	146	40.27	31.77	8.81 (7.09, 10.53)	<0.001	0.00
LVEDD (mm/m^2^)	1	33	58	30.00	30.00	0.00 (−1.02, 1.02)	>0.99	N/A
LVSV (ml/m^2^)	3	41	36	62.61	51.74	11.60 (8.79, 14.40)	<0.001	0.00
LVEF (%)	5	125	152	60.82	61.70	−1.16 (−2.44, 0.12)	0.075	0.00
LVM (g/m^2^)	4	112	139	57.32	42.85	14.11 (8.65, 19.57)	<0.001	0.86
LVISWT (mm/m^2^)	1	33	58	4.90	4.50	0.40 (0.13, 0.67)	0.004	N/A
RVEDV (ml/m^2^)	5	125	152	112.01	90.08	20.69 (15.65, 25.74)	<0.001	0.57
RVESV (ml/m^2^)	4	117	146	49.75	38.55	10.66 (8.51, 12.81)	<0.001	0.00
RVEDD (mm/m^2^)	1	33	58	23.00	23.00	0.00 (−1.45, 1.45)	>0.99	N/A
RVSV (ml/m^2^)	3	41	36	62.66	50.01	11.58 (7.47, 15.70)	<0.001	0.26
RVEF (%)	5	125	152	54.90	56.84	−1.60 (−2.88, −0.31)	0.015	0.00
RVM (g/m^2^)	4	112	139	17.23	13.25	3.71 (2.17, 5.25)	<0.001	0.79
Echocardiography								
LVSV (ml/m^2^)	5	146	136	54.34	47.11	7.17 (1.36, 12.98)	0.016	0.90
LVEDV (ml/m^2^)	6	166	156	72.89	61.01	12.22 (7.99, 16.45)	<0.001	0.79
LVESV (ml/m^2^)	4	111	105	30.25	22.08	8.09 (4.90, 11.27)	<0.001	0.86
LVEDD (mm/m^2^)	7	189	189	28.37	27.73	0.72 (0.17, 1.26)	0.010	0.44
LVESD (mm/m^2^)	2	55	55	18.60	17.85	0.74 (0.11, 1.37)	0.022	0.00
LVM (g/m^2^)	8	218	193	83.65	63.70	19.76 (11.11, 28.41)	<0.001	0.94
LVPWT (mm/m^2^)	7	195	184	5.06	4.35	0.72 (0.42, 1.02)	<0.001	0.86
IVSWT (mm/m^2^)	8	241	232	5.33	4.66	0.71 (0.44, 0.97)	<0.001	0.95
LVEF (%)	7	212	204	59.93	61.86	−1.75 (−4.50, 1.01)	0.21	0.91
RVSV (ml/m^2^)	3	65	55	47.53	37.67	9.49 (5.15, 13.84)	<0.001	0.81
TAPSE (mm)	3	81	85	23.30	24.30	−1.17 (−3.29, 0.96)	0.28	0.78
RV FAC (%)	1	33	37	49.40	46.00	3.40 (−0.25, 7.05)	0.068	N/A
RVEDA (cm/m^2^)	4	121	121	11.50	9.97	1.50 (0.50, 2.50)	0.003	0.83
RVESA (cm/m^2^)	2	66	70	5.43	5.05	0.39 (−0.83, 1.60)	0.53	0.93
RVEDV (ml/m^2^)	2	45	35	83.55	59.60	23.60 (9.80, 37.40)	0.001	0.86
RVESV (ml/m^2^)	2	45	35	37.10	23.00	13.87 (8.20, 19.53)	<0.001	0.70
RVEF (%)	2	45	35	56.45	61.25	−4.92 (−7.03, −2.82)	<0.001	0.00

Note

*p*‐Values result from random effects models. *I* ^2^ is undefined when there is only one study.

Abbreviations: BSA, body surface area; CI, confidence interval; LVEDD, left ventricular end‐diastolic diameter index to BSA (mm/m^2^); LVEDV, left ventricular end‐diastolic volume indexed to BSA (ml/m^2^); LVEF, left ventricular ejection fraction (%); LVESD, left ventricular end‐systolic volume index to BSA (ml/m^2^); LVISWT, left ventricular inferior septal wall thickness index to BSA (mm/m^2^); LVM, left ventricular mass index to BSA (g/m^2^); LVPWT, left ventricular posterior wall thickness index to BSA (mm/m^2^); LVSV, left ventricular stroke volume index to BSA (ml/m^2^); LVSWT, left ventricular septal wall thickness index to BSA (mm/m^2^); RV FAC, right ventricular fractional area change (%); RVEDA, right ventricular end‐diastolic area indexed to BSA (cm/m^2^); RVEDD, right ventricular end‐diastolic diameter index to BSA (mm/m^2^); RVEDV, right ventricular end‐diastolic volume index to BSA (ml/m^2^); RVEF, right ventricular ejection fraction (%); RVESA, right ventricular and systolic area index to BSA (cm/m^2^); RVESV, right ventricular end‐systolic volume index to BSA (ml/m^2^); RVM, right ventricular mass index to BSA (ml/m^2^); RVSV, right ventricular stroke volume index due to BSA (ml/m^2^); TAPSE, tricuspid valve systolic excursion (mm).

### 3.1 CMR analysis

When assessed using CMR, LVEDV (Kramer et al., 2013; Luijkx, Cramer, et al., 2012; Luijkx, Velthuis, et al., 2012; Mangold et al., 2013; Petersen et al., 2006; Prakken et al., 2011; Steding‐Ehrenborg et al., 2016) (+17.0%; 102.1 vs. 84.7 ml/m^2^; *p* < 0.001), LVSV (Kramer et al., 2013; Mangold et al., 2013; Petersen et al., 2006; Steding‐Ehrenborg et al., 2016) (+18.5%, 62.6 vs. 51.7 ml/m^2^; *p* < 0.001), and LVM (Luijkx, Cramer, et al., 2012; Petersen et al., 2006; Prakken et al., 2010; Steding‐Ehrenborg et al., 2016) (+25.0%, 57.3 vs. 42.8 g/m^2^; *p* < 0.001) were greater in EA versus HC. LVEF tended to be ~2% lower in EA versus HC (60.8% vs. 61.7%; *p* = 0.075).

**FIGURE 2:**
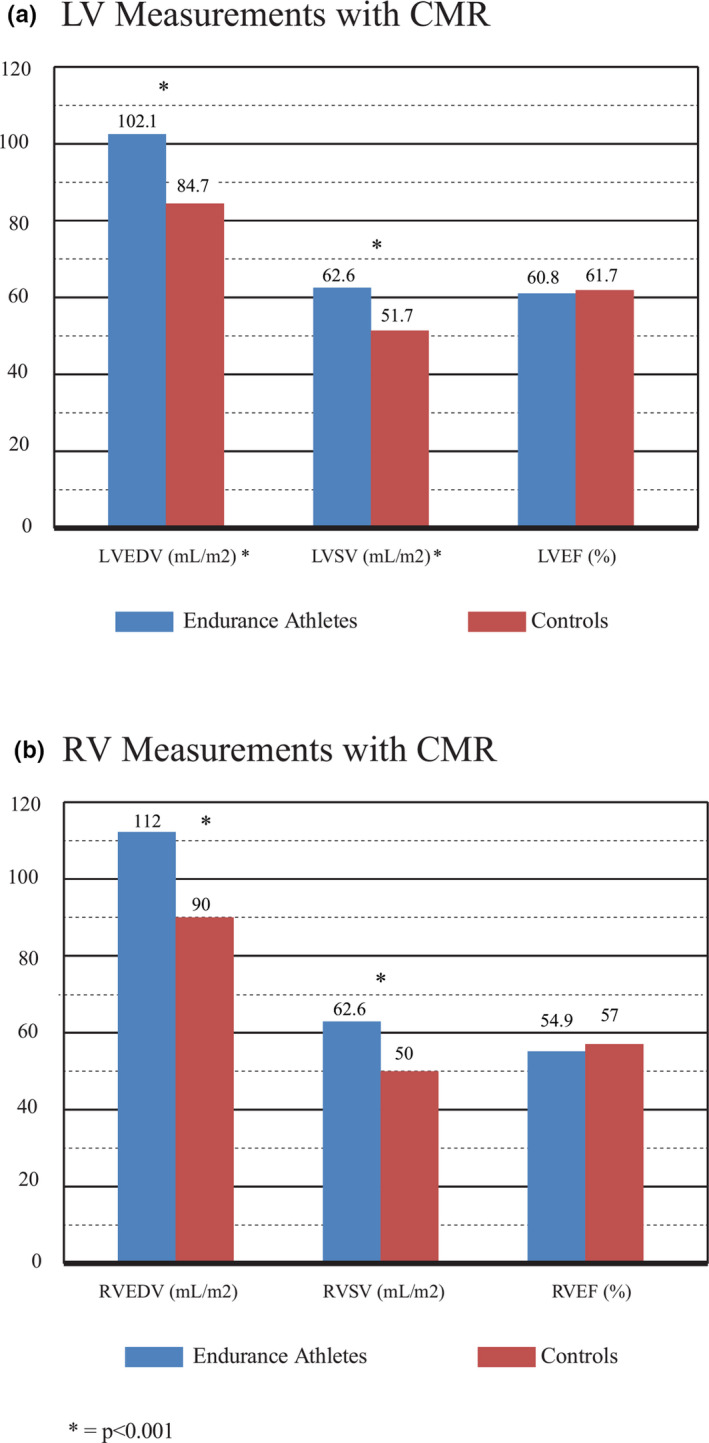
Bar graph showing mean values for LVEDV indexed to BSA (ml/m^2^), LVSV indexed to BSA (ml/m^2^), and LVEF (%) (a) for EA and HCs obtained with CMR. Mean values for RVEDV indexed to BSA (ml/m^2^), RVSV indexed to BSA (ml/m^2^), and RVEF (%) (b) for EA and HCs using CMR (b). Statistically significant differences are indicated with an asterisk (*). BSA, body surface area; CMR, cardiac magnetic resonance imaging; LVEDV, left ventricular end‐diastolic volume; LVEF, left ventricular ejection fraction; LVSV, left ventricular stroke volume; RVEDV, right ventricular end‐diastolic volume; RVEF, right ventricular ejection fraction; RVSV, right ventricular stroke volume

Assessment of RV function via CMR revealed an 18.5% greater RVEDV (Kramer et al., 2013; Luijkx, Cramer, et al., 2012; Luijkx, Velthuis, et al., 2012; Mangold et al., 2013; Petersen et al., 2006; Prakken et al., 2010; Steding‐Ehrenborg et al., 2016) (112.0 vs. 90.1 ml/m^2^; *p* < 0.001), an 18.5% greater RVSV (Kramer et al., 2013; Mangold et al., 2013; Petersen et al., 2006; Steding‐Ehrenborg et al., 2016) (62.7 vs. 50.0 ml/m^2^; *p* < 0.001), and a 22% greater RVM (Luijkx, Cramer, et al., 2012; Petersen et al., 2006; Prakken et al., 2010; Steding‐Ehrenborg et al., 2016) (17.2 vs 13.3 g/m^2^; *p* < 0.001) in EA versus HC. RVEF (Kramer et al., 2013; Luijkx, Cramer, et al., 2012; Luijkx, Velthuis, et al., 2012; Mangold et al., 2013; Petersen et al., 2006; Prakken et al., 2010; Steding‐Ehrenborg et al., 2016) was ~2% lower in EA versus HC (54.9% vs. 56.8%; *p* < 0.015). A graphical representation of differences in LVEDV and RVEDV is shown in Figure 2. Additional CMR measurements are included in Table 2.

### 3.2 Echocardiographic assessment

Transthoracic echocardiography assessment of LV size revealed a 17% greater LVEDV (Doronina et al., 2018; Kooreman et al., 2019; Lakatos et al., 2018; Leischik & Spelsberg, 2014; Leischik et al., 2016; Malmgren et al., 2015; Sanz‐de la Garza et al., 2017; Zeldis et al., 1978) (72.9 vs. 61.0 ml/m^2^; *p* < 0.001) and a 27% greater LVESV (Doronina et al., 2018; Lakatos et al., 2018; Leischik & Spelsberg, 2014; Leischik et al., 2016; Malmgren et al., 2015) in EA versus HC (30.3 vs. 22.1 ml/m^2^; *p* < 0.001). LVEF (Doronina et al., 2018; Hedman et al., 2015; Kooreman et al., 2019; Lakatos et al., 2018; Leischik & Spelsberg, 2014; Leischik et al., 2016; Malmgren et al., 2015; Venckunas et al., 2016) tended to be lower by 3% in EA versus HC (59.9% vs. 61.9%; *p* = 0.21). Assessment of RV function revealed a 28% greater RVEDV (Doronina et al., 2018; Lakatos et al., 2018) (83.5 vs. 59.6 ml/m^2^; *p* < 0.001) and a 37% greater RVESV (Doronina et al., 2018; Lakatos et al., 2018) in EA versus HC (37.1 vs. 23.0 ml/m^2^; *p* < 0.001). RVEF (D’Ascenzi et al., 2017; Doronina et al., 2018; Lakatos et al., 2018) was reduced by 9% in EA versus HC (56.5% vs. 61.3%; *p* < 0.001).

Few studies reported data for the MA population, therefore a limited analysis was performed on the MA to HC population and this information is provided in Appendix S2. A limited comparison of EA and MA was also performed and can be found in Appendix S3. Due to limited data across the spectrum of variables, a statistical analysis on SA was not conducted in this study.

## 4 DISCUSSION

**FIGURE 3:**
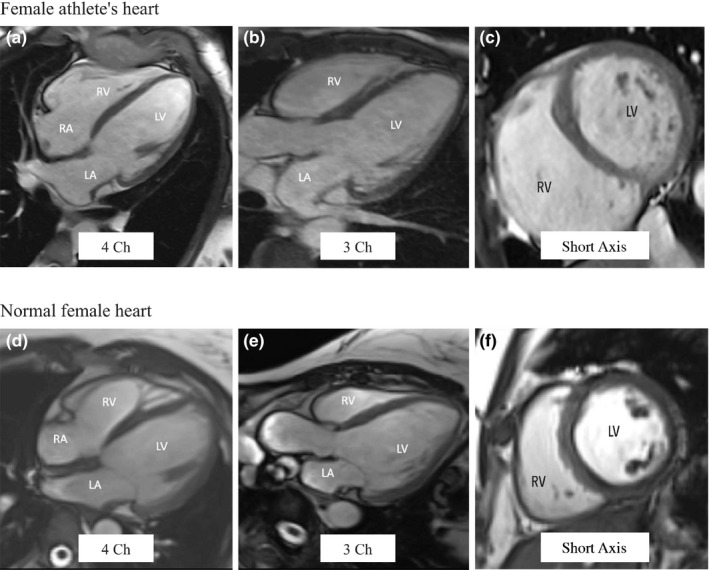
CMR images obtained from an elite female athlete and a normal healthy female. Images obtained from an elite female athlete include four chamber (a), three chamber (b), and short axis (c) views. Measurements obtained are as follows: LVEDV 102 ml/m^2^, LVESV 39 ml/m^2^, LVSV 63 ml/m^2^, and LVEF 62%, RVEDV 116 ml/m^2^, RVESV 47 ml/m^2^, RVSV 69 ml/m^2^, RVEF 59%. A CMR from a healthy female is used for visual comparison which include four chamber (d), three chamber (e), and short axis (f) views. Measurements obtained are as follows: LVEDV 80 ml/m^2^, LVESV 35 ml/m^2^, LVSV 46 ml/m^2^, and LVEF 57%, RVEDV 67 ml/m^2^, RVESV 26 ml/m^2^, RVSV 41 ml/m^2^, RVEF 59%. All values not represented with a % are indexed to BSA. This figure highlights differences in ventricular size between female athletes and healthy controls. 2 Ch =2 chamber; 3 Ch =3 chamber; 4 Ch =4 chamber. BSA, body surface area; CMR, cardiac magnetic resonance imaging; LV, left ventricle; LVEDV, left ventricular end‐diastolic volume; LVESV, left ventricular end‐systolic volume; LVEF, left ventricular ejection fraction; LVSV, left ventricular stroke volume; RV = right ventricle; RVEDV, right ventricular end‐diastolic volume; RVESV, right ventricular end‐systolic volume; RVEF, right ventricular ejection fraction; RVSV, right ventricular stroke volume

This evidence‐synthesis review summarized the evidence of biventricular size and function in female athletes. Findings demonstrated marked changes in ventricular size and modest changes to function in a manner similar to that of male athletes (D'Ascenzi et al., 2017, 2018; Morganroth et al., 1975; Pelliccia et al., 1991, 1999; Utomi et al., 2013). When assessed using CMR and TTE, both LV and RV volumes and strokes volumes were statistically greater (*p* < 0.001) in EA compared to HC. Although not statistically significant, there was a trend toward a lower LVEF in EA compared to HC when assessed with both CMR and TTE. The same was true when assessing the RV with CMR with a lower RVEF in EA versus HC (*p* = 0.015). However, TTE‐ derived RVEF was lower in EA compared to HC (*p* < 0.001). Figure 3 provides a comparison of CMR images obtained from an elite female athlete and a normal healthy female as an example of differences seen between these groups.

The demands placed on the cardiovascular system by elite EA lead to larger ventricular volumes and masses (Kawel‐Boehm et al., 2015; Maron, 1986; Maron & Pelliccia, 2006; Morganroth et al., 1975; Pelliccia et al., 1999; Pluim et al., 2000; Rawlins et al., 2009). At rest, lower EFs may be seen in athletes compared to controls, however, this is countered by larger ventricular volumes, SVs, and enhanced tissue oxygen extraction (Abergel et al., 2004; Baggish & Wood, 2011; Gilbert et al., 1977; Heinonen et al., 2014). This higher SV permits increased cardiac output and blood transit time during exercise, enabling tissues to extract more oxygen and perform to a greater capacity (Baggish & Wood, 2011; Heinonen et al., 2014).

Interestingly, structural changes in EA revealed a disproportionate increase in RV volumes compared to LV volumes when measured by both CMR and TTE, with a more pronounced difference with TTE assessment. This finding suggests that the thin walled RV may be more susceptible to dilation when exposed to repeated bouts of exercise than the thicker walled LV (La Gerche et al., 2017). In addition, TTE‐derived LVEF was 3% lower in EA versus HC, whereas RVEF was 9% lower in EA versus HC. Based on this trend, one would expect a similar reduction in TAPSE and RV FAC. However, the actual reported results are equivocal, with EA exhibiting an overall reduction in TAPSE (mean difference −1.17 mm) and an increase in RV FAC (mean difference 3.40%) when compared to HC. An explanation for the aforementioned finding is unclear, but may reflect the challenges of assessing the RV using echocardiography (La et al., 2013; Grothues et al., 2002, 2004). Another explanation may be related to the small number of studies reporting RV volumetric data by echocardiographic assessments. Additionally, assessment of RVEF with CMR revealed only a modest 2% reduction in EA versus HC. Therefore, CMR and TTE findings are consistent and point to the need for additional data on RV function in this cohort. When comparing values obtained from different imaging modalities, it is important to highlight consistently increased volumetric measurements in EA and HC obtained with CMR. CMR provides superior spatial resolution and a more accurate functional assessment due to better visualization of ventricular borders and cavity assessment from apex to base (Gardner et al., 2009; Marwick et al., 2013). Volumetric assessment of ventricular cavities with TTE may mistake trabeculations as myocardium rather than part of the LV cavity, creating underestimations in measurements (Mor‐Avi et al., 2008). CMR also allows better visualization and measurement of the base. As a result, CMR has superior interobserver reproducibility compared to two‐dimensional (2D) echocardiography (Grothues et al., 2002; Marwick et al., 2013).

The first systematic review evaluating the male athlete's heart was published in 2000 and used two‐dimensional echocardiography exclusively to focus on morphological changes to the LV with respect to sport category, postulating that divergent cardiac adaptations exist between endurance and strength sporting disciplines (Pluim et al., 2000). While this study could not make explicit correlations of EA exhibiting more eccentric changes and strength‐trained athletes possessing more concentric changes, it did note increased left ventricular end‐diastolic internal diameters across all athlete groups in comparison to controls, supporting findings from earlier studies (Fagard, 1996; Morganroth et al., 1975; Pelliccia et al., 1991, 1999). A follow‐up meta‐analysis in 2013 incorporated CMR for cardiac structural assessment (Utomi et al., 2013). The evidence presented in this systematic review supported an “eccentric‐type” hypertrophy and reported upper limits for chamber dimensions in EA. Importantly, neither study reported data specifically for female athletes. Although a meta‐analysis was performed on female athletes in 2004, data were limited to the LV obtained with TTE and showed increased LV internal diameter and LVM regardless of sporting category (Whyte et al., 2004). Another systematic review and meta‐analysis by D'Ascenzi et al. (2017) evaluated reference values for the right heart in competitive athletes. Unfortunately, the small sample size of females limited a comprehensive analysis of right heart function to TTE and not CMR which may have provided a more complete assessment of right heart structure and function. The most recent systematic review and meta‐analysis again focused on the male athlete's heart using CMR to derive reference values for biventricular size and function according to sport category, and found the upper limits of biventricular dimensions to be higher in comparison to the general population (D’Ascenzi et al., 2018). This study also found a pronounced increase in RV dimensions with many athletes meeting diagnostic criteria for ARVC based on European guidelines (Marcus et al., 2010).

While there are limited number of female‐specific studies evaluating such changes, the majority of them noted similar findings to this present review. One study on the female athlete's heart reported lower absolute volumes and masses in female athletes when compared to their male counterparts (Petek & Wasfy, 2018). However, when these values were indexed to BSA, there was a paradoxical reversal with female values being greater than males. Another study on Italian Olympic Athletes evaluated 600 female competitors using TTE and found increased LVEDV and LV wall thickness with 1% of contestants meeting diagnostic criteria for dilated cardiomyopathy (Pelliccia et al., 1996).

Ultimately, our literature search resulted in a limited number of studies reporting values for the two other major sport categories; mixed and strength disciplines. Therefore, an analysis of cardiac changes was not thoroughly evaluated in these populations. While data were sufficient to perform a meta‐analysis with EA and HC, this information is still limited due to sample size. Additional limitations include data from non‐comparative studies and heterogeneity in data regarding sport category and assessment methods. This is the first systematic review that synthesizes evidence to answer questions about cardiac structural and functional changes specifically in female athletes. Additionally, this work derives strength from its methodological approach and thorough literature search. Further studies are needed to determine if there are potential adverse outcomes secondary to such changes in this population.

## 5 CONCLUSIONS

Structural and functional characteristics of the “athlete's heart” have been well defined for male athletes with the most notable differences being chamber enlargement. Based on the data presented in the present review, similar findings hold true for female athletes, with the most notable observation being bi‐ventricular enlargement in EA. There was a trend for a disproportionate increase in RV over LV volumes. Additionally, volumetric assessment tended to be dependent on imaging modality. Overall, larger volumetric measurements were obtained with CMR. However, there were more pronounced differences between in LV and RV measurements in EA and HC when using TTE. Because such differences exist between different imaging modalities, future studies assessing the RV with exercise CMR may provide a more accurate assessment of RV structure and function in this subject population. Additionally, exercise CMR may also shed light on potential pathologic adaptations when the RV is exposed to repeated bouts of intense exercise. Although there are no reference standards for ventricular structure and function in female athletes, it is important to acknowledge that differences exist between HC and athletes when interpreting data in the clinical setting, as some physiologic changes may resemble pathologic processes. The quality of evidence was generally low, mainly due to indirectness and limitation in study design (overall higher risk of bias).

## 6 CLINICAL COMPETENCIES

This work will advance providers medical knowledge with regards to normative structural and functional values when assessing female athlete's hearts with TTE or CMR.

## 7 TRANSLATIONAL OUTLOOK

The primary impediment with performing this study was the limited data available on TTE and CMR analysis of the female athlete's heart. Therefore, further investigations are necessary in order to develop standard reverence values with TTE and CMR imaging in this population. Additionally, advanced imaging techniques such as exercise CMR may define adaptations in the RV and LV undergo when stressed to potentially identify potential pathological adaptations.

## FUNDING INFORMATION

There was no funding for this paper.

## CONFLICT OF INTEREST

None declared.

## AUTHOR CONTRIBUTIONS

The authors confirm contribution to the paper as follows: study conception and design: Robyn Bryde, Andres Applewhite, Brian Shapiro, and M. Abu Dabrh; data collection: Robyn Bryde, Andres Applewhite, Larry Prokop, Tara Brigham, Michael Heckman, and Carlos Rojas; analysis and interpretation of results: M. Abu Dabrh, Robyn Bryde, Andres Applewhite, and Brian Shapiro; draft manuscript preparation: Bryan Taylor, Sara Filmalter, George Pujalte, Alexander Heckman, Brian Shapiro, and Robyn Bryde. All authors have reviewed the results and approved the final manuscript.

## Supporting information



Appendix S1

Appendix S2

Appendix S3

Appendix S4a

Appendix S4b
