# Supplementary figures and images for: Cardiac structure and function in elite female athletes: A systematic review and meta‐analysis
Source: Physiol Rep. 2021 Dec 11;9(23):e15141. doi: 10.14814/phy2.15141 (PMC8665377; doi:10.14814/phy2.15141)

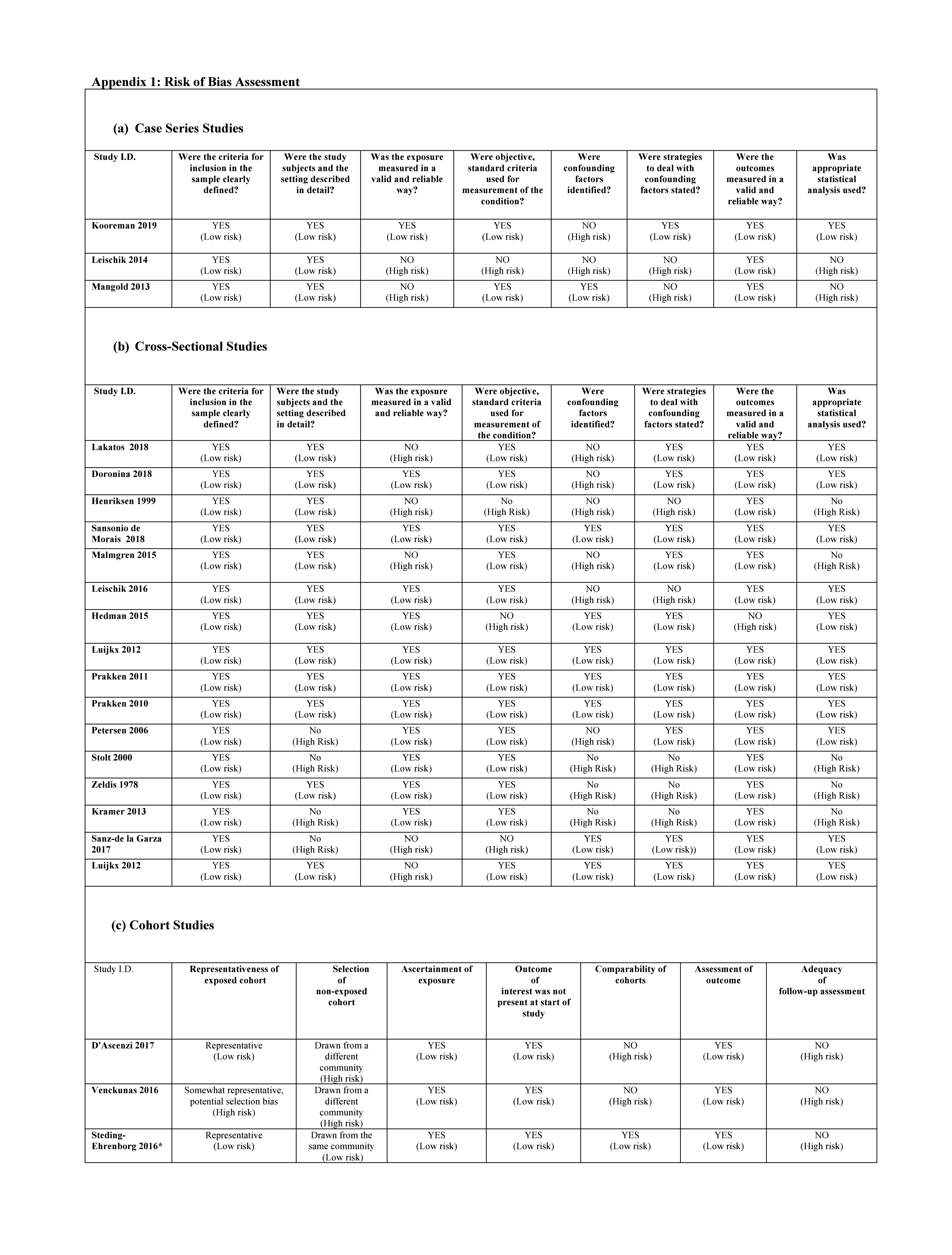

Supplement: Supplementary file 1 — Appendix S1 [file PHY2-9-e15141-s002.png]

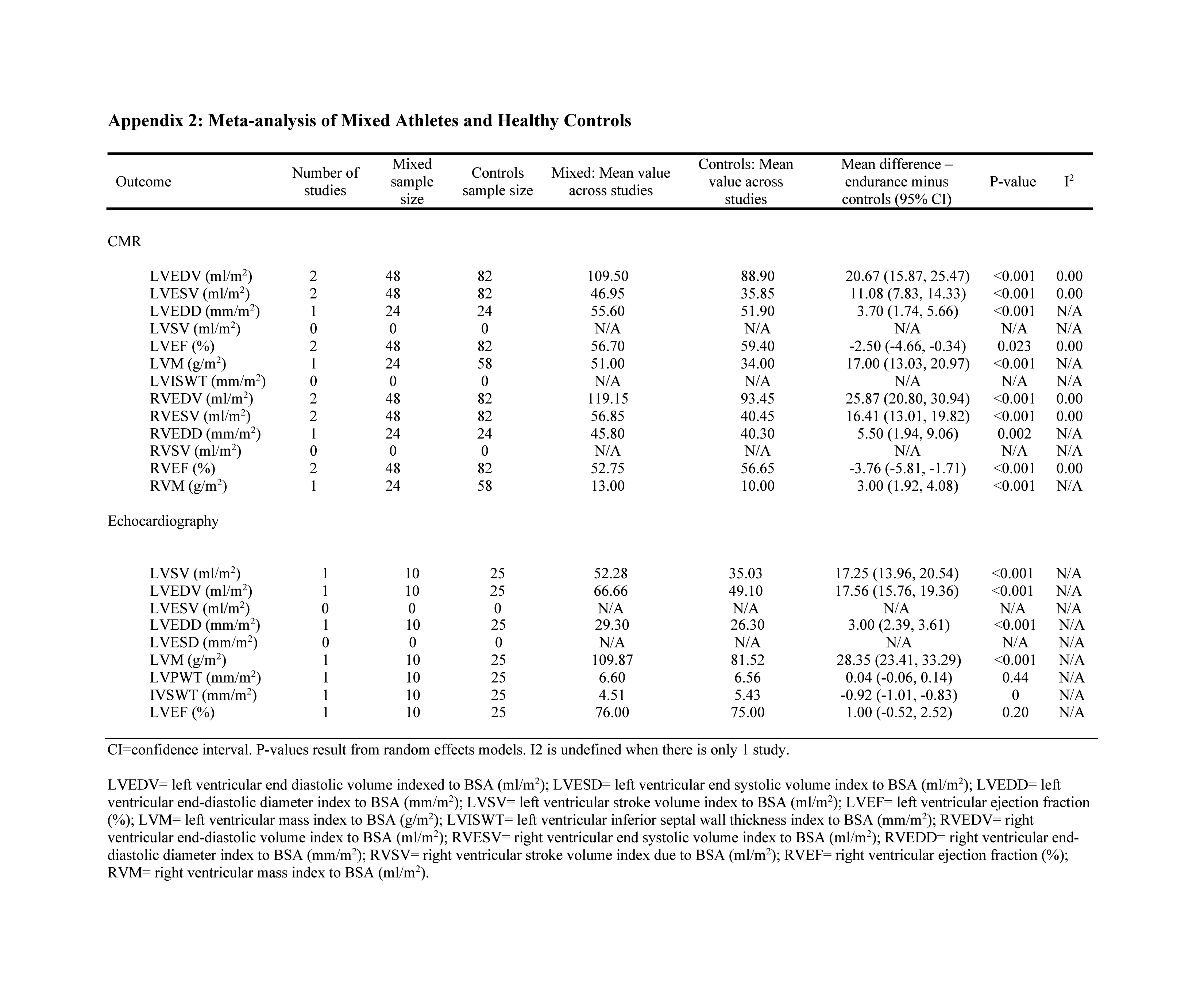

Supplement: Supplementary file 2 — Appendix S2 [file PHY2-9-e15141-s004.png]

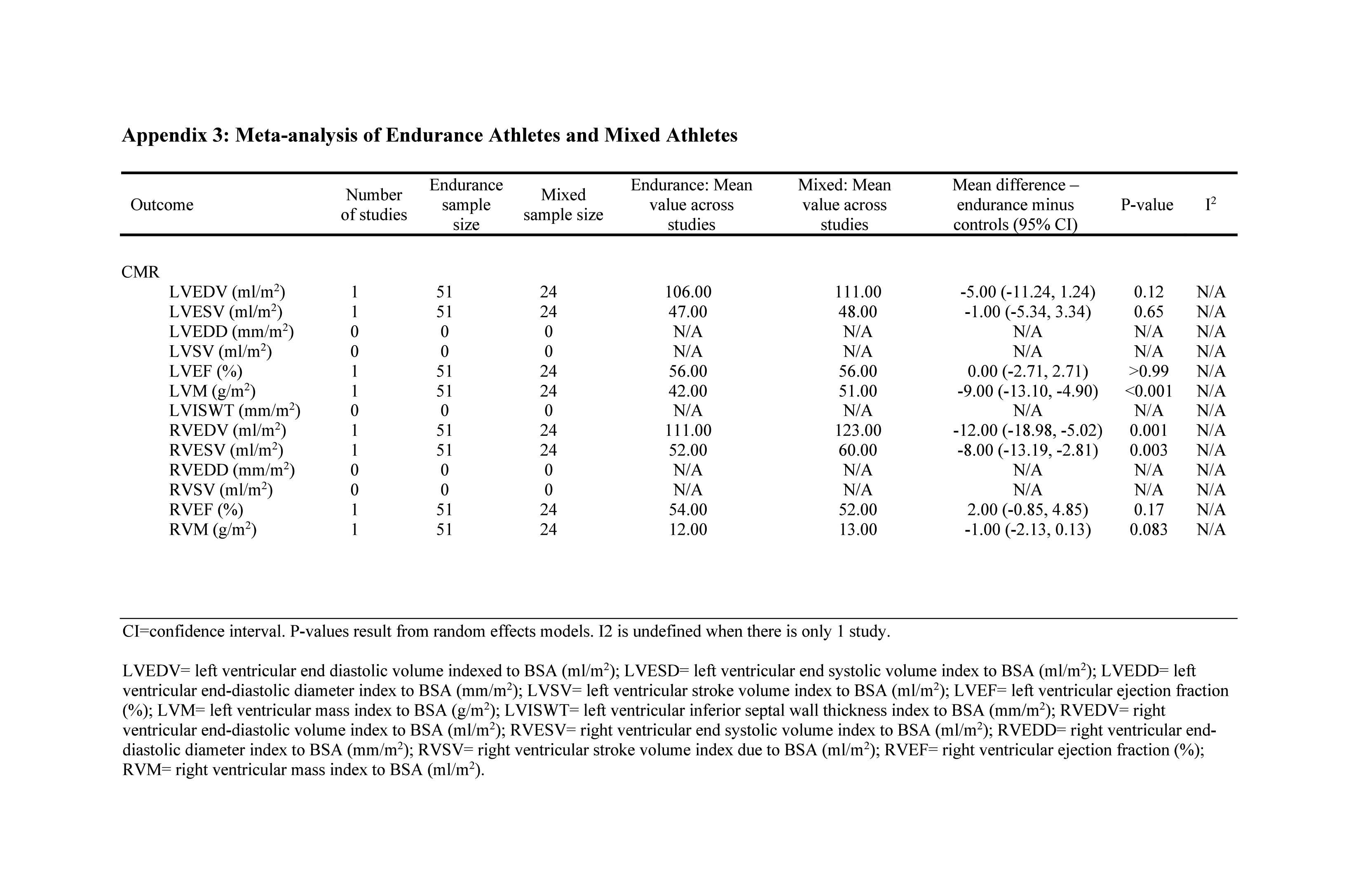

Supplement: Supplementary file 3 — Appendix S3 [file PHY2-9-e15141-s001.png]

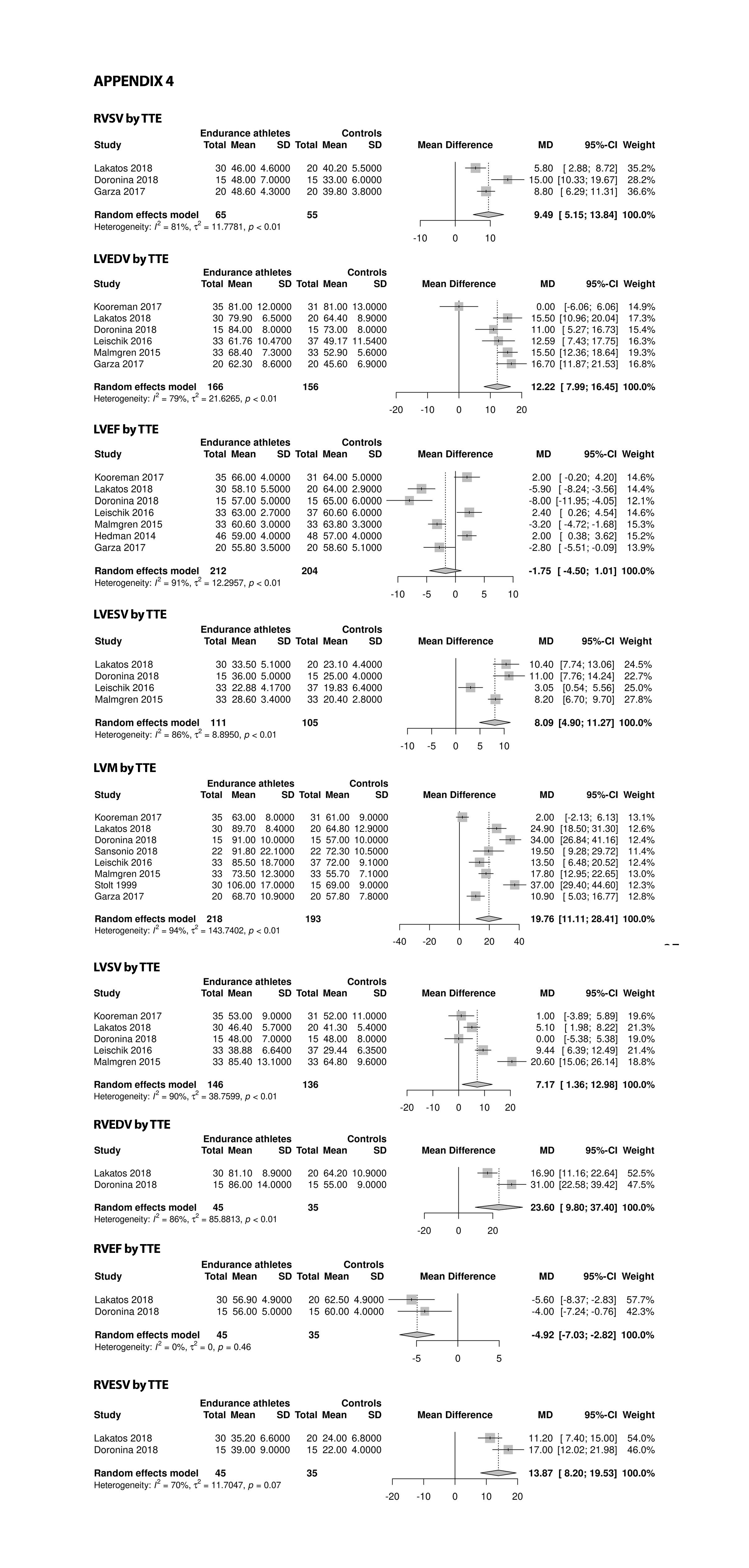

Supplement: Supplementary file 4 — Appendix S4a [file PHY2-9-e15141-s003.png]

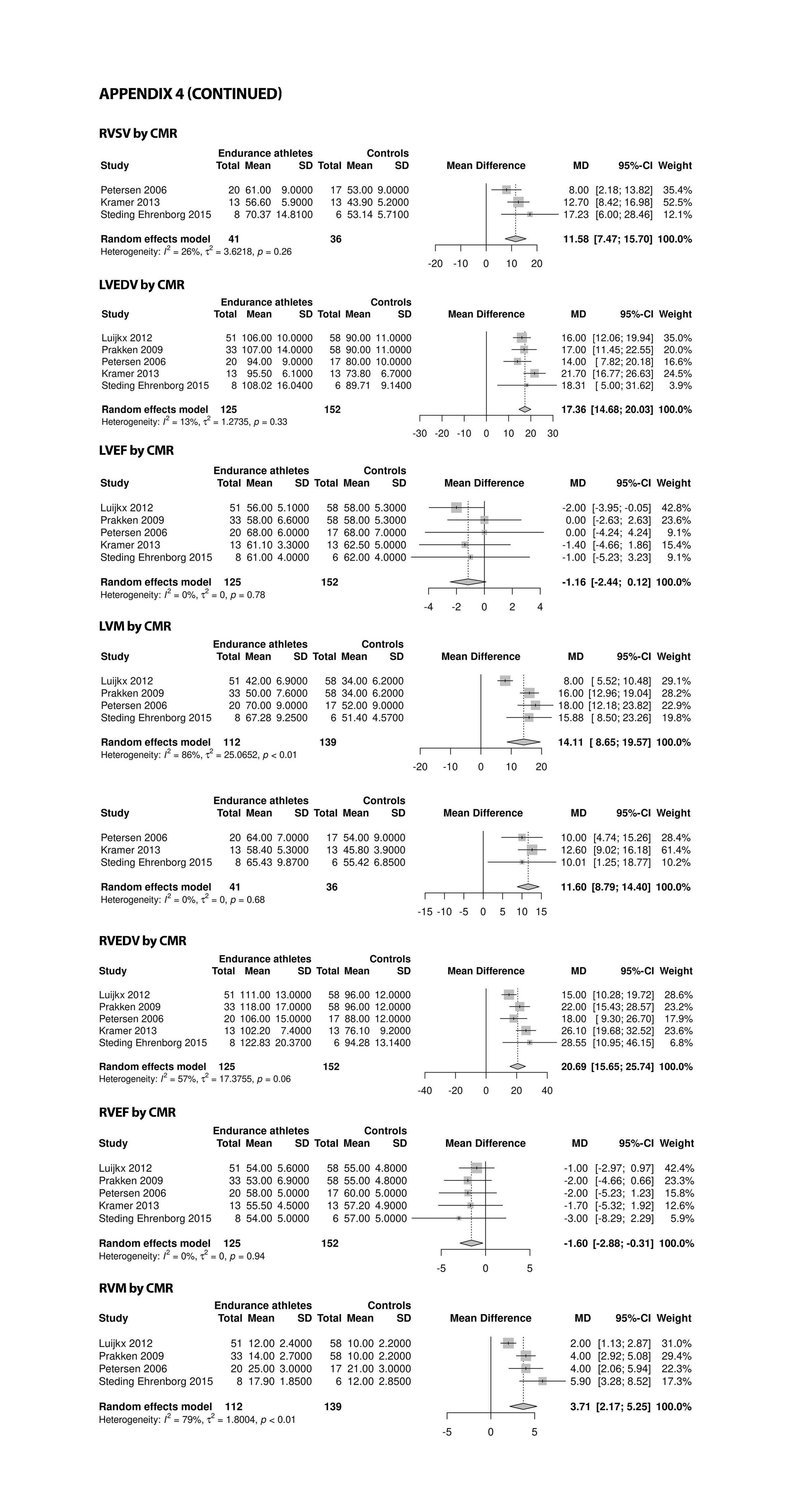

Supplement: Supplementary file 5 — Appendix S4b [file PHY2-9-e15141-s005.png]
